# Thermal–Inflammatory Index (TI): An Integrated Biomarker of Severity and Prognosis in Chronic Lower-Limb Ulcers

**DOI:** 10.3390/biomedicines14030680

**Published:** 2026-03-16

**Authors:** Bartosz Molasy, Małgorzata Wrzosek

**Affiliations:** 1 Department of Surgical Medicine, Medical College, Jan Kochanowski University, 25-317 Kielce, Poland; 2 Department of General Surgery and Coloproctology, St Alexander Hospital, 25-316 Kielce, Poland; 3 Department of Pharmaceutical Sciences, Medical College, Jan Kochanowski University, 25-317 Kielce, Poland; malgorzata.wrzosek@wum.edu.pl; 4 Department of Biochemistry and Pharmacogenomics, Medical University of Warsaw, 02-097 Warsaw, Poland

**Keywords:** thermography, chronic wounds, inflammation, biomarkers, risk stratification, lower-limb ulcers, prognosis

## Abstract

**Background/Objectives**: Chronic lower-limb ulcers of mixed etiology are characterized by impaired microcirculation and persistent inflammation, leading to delayed healing, frequent hospitalizations, and a high risk of limb loss. While infrared thermography reflects local perfusion status and systemic inflammatory markers capture whole-body immune activation, these dimensions are usually assessed separately. The objective of this study was to develop and internally evaluate a composite Thermal–Inflammatory Index (TI) integrating wound-bed thermography with systemic inflammatory markers to stratify disease severity and prognosis in patients with chronic lower-limb ulcers. **Methods**: In this prospective observational study, 82 adults with chronic lower-limb ulcers underwent baseline infrared thermographic assessment of wound-bed temperature using a standardized protocol. Concurrently, neutrophil-to-lymphocyte ratio (NLR) and C-reactive protein (CRP) were measured. The Thermal–Inflammatory Index was constructed as a standardized composite of inverted wound-bed temperature, NLR, and CRP. A simplified TI score (0–3) was derived using predefined clinical thresholds. The primary endpoint was a composite adverse outcome defined as amputation or failure to achieve complete wound healing within 12 weeks. Secondary outcomes included a prolonged hospital stay (>7 days). Discriminative performance was assessed using receiver operating characteristic analysis, and associations were examined using correlation and logistic regression models. **Results**: Higher TI values were associated with colder wound beds, elevated systemic inflammatory markers, and increased disease burden. The TI demonstrated moderate discrimination for the composite adverse outcome (AUC 0.75) and prolonged hospitalization (AUC 0.71), performing comparably to the strongest single component (−T_bed, AUC 0.77) while integrating local and systemic information. Each one-standard-deviation increase in TI was independently associated with higher odds of the composite adverse outcome and a prolonged hospital stay. The simplified TI score showed clear stepwise gradients in adverse outcomes and length of hospitalization. **Conclusions**: The Thermal–Inflammatory Index integrates thermographic and inflammatory signals into a single, clinically interpretable biomarker of severity and prognosis in chronic lower-limb ulcers. TI and the simplified TI score may support early risk stratification using low-cost, bedside-accessible data.

## 1. Introduction

Chronic lower-limb ulcers of mixed etiology—including diabetic, ischemic, mixed arterial–venous, venous and traumatic ulcers—represent a significant clinical and healthcare burden. Estimates from population-based studies suggest that 1–2% of adults in high-income countries live with a chronic wound, with prolonged healing trajectories, recurrent infections, impaired mobility and frequent hospitalizations contributing to morbidity and healthcare expenditure [1,2]. Although the underlying causes differ, many of these ulcers share common pathophysiological mechanisms, particularly microcirculatory impairment and chronic inflammation [1,2,3,4].

Microvascular dysfunction restricts the delivery of oxygen and nutrients required for tissue repair, whether due to macrovascular disease, microangiopathy, venous hypertension, edema or structural deformities of the limb [2,3,4]. In parallel, chronic activation of innate and adaptive inflammatory pathways leads to excessive protease activity, oxidative stress and ineffective extracellular matrix turnover, trapping the wound in a state of non-resolving inflammation [1,2,5]. This combination of perfusion impairment and inflammatory dysregulation contributes to adverse outcomes across ulcer etiologies.

Despite this shared biology, routine assessment of chronic lower-limb ulcers remains largely descriptive. Clinicians rely on visual inspection, episodic photography, ankle-brachial or toe-pressure measurements when available, and a small number of systemic inflammatory markers. Severity scores and systemic inflammatory response syndrome (SIRS) criteria have been studied primarily in diabetic foot infection but are applied broadly to mixed ulcer presentations, although they reflect systemic illness rather than the microenvironment of the wound [6,7]. Likewise, laboratory indices such as the neutrophil-to-lymphocyte ratio (NLR) and C-reactive protein (CRP) have been associated with delayed healing, amputation and poor outcomes—findings derived largely from diabetic and peripheral arterial disease cohorts but grounded in pathophysiological processes common to many chronic ulcers [8,9,10,11,12,13]. These markers describe systemic inflammatory tone but provide no direct insight into local perfusion or thermal patterns at the wound surface.

Management of chronic wounds is typically multidisciplinary and includes pressure offloading, debridement, infection control, optimization of glycemic status and comorbidities, and restoration of perfusion when indicated (e.g., revascularization) [14]. Systemic antibiotic therapy is frequently initiated in hospitalized patients, yet microbiological heterogeneity and the growing burden of multidrug-resistant (MDR) organisms complicate empirical treatment and may delay targeted therapy [15]. These challenges increase resource utilization and contribute to prolonged hospitalization and risk escalation [15]. Simple bedside tools that help identify high-risk physiological phenotypes may therefore support earlier escalation, rational antimicrobial stewardship, and timely referral for vascular evaluation.

Infrared thermography has emerged as a promising method to capture local physiological signals at the bedside. A comprehensive scoping review of point-of-care thermographic devices highlighted their utility across diverse wound types—burns, venous ulcers, diabetic ulcers, pressure injuries and postoperative wounds—where thermal imaging can detect early inflammation, infection, tissue compromise and altered perfusion [16]. Additional reviews emphasize that superficial temperature distributions reflect cutaneous blood flow and metabolic activity, and that chronic ulcers frequently exhibit characteristic patterns such as a relatively “cold” wound bed or altered temperature gradients between the ulcer and surrounding tissue [17,18,19,20]. These patterns correlate with impaired healing and risk of deterioration, whereas periwound hyperthermia may indicate active inflammation or infection [17,18,19,20]. However, thermographic use in clinical practice remains heterogeneous, with inconsistent acquisition protocols, variable regions of interest and a lack of validated quantitative indices that could support decision-making [16,17,18,19].

More granular biomarker work has focused on the biochemical composition of wound exudate, identifying cytokines, proteases and neuropeptides associated with pain, chronicity and healing trajectories [21]. Although mechanistically informative, these assays require specialized sampling and laboratory platforms and are unsuitable for wide clinical implementation. By contrast, NLR and CRP are universally available, inexpensive and repeatedly associated with poor outcomes across high-risk wound populations, reflecting a systemic inflammatory response that often accompanies microvascular dysfunction [8,9,10,11,12,13]. Local thermographic patterns encode information about perfusion and the wound microenvironment, while systemic inflammatory indices such as NLR and CRP capture whole-body immune activation and vascular stress [10,11,12,13,16,19]. Both dimensions are relevant across ulcer etiologies, but they are typically evaluated in isolation.

Although multiple studies have examined thermography and inflammatory markers separately, integrated quantitative approaches remain limited. To date, no study has quantitatively integrated thermal wound-bed information with systemic inflammatory markers into a single biomarker of severity and prognosis for chronic lower-limb ulcers of mixed etiology. This gap limits the clinical applicability of available physiological information. Clinicians require simple, reproducible tools that reflect the combined burden of microcirculatory failure and systemic inflammation and can be applied rapidly at the bedside or in ambulatory care. Motivated by this need, we developed the Thermal–Inflammatory Index (TI), a continuous composite measure incorporating wound-bed temperature, NLR and CRP, along with a simplified TI score (0–3) based on binary thresholds for each component. We evaluated the distribution, discriminative performance and bedside applicability of TI and the TI score in a prospective cohort of patients with mixed-etiology chronic lower-limb ulcers.

## 2. Materials and Methods

### 2.1. Study Design

This was a single-center, prospective observational study evaluating infrared thermography and systemic inflammatory markers in patients presenting with chronic lower-limb ulcers. The primary objective of the present analysis was to develop and internally evaluate a novel composite Thermal–Inflammatory Index (TI) integrating local thermal characteristics of the wound with the systemic inflammatory response. Data were collected consecutively at hospital admission and during treatment. The study followed the STROBE reporting guidelines for observational research.

### 2.2. Study Population

All adults aged 18 years or older admitted with a diagnosis of chronic lower-limb ulcer were screened for eligibility. A chronic ulcer was defined as a non-healing, full-thickness defect of the foot, ankle, or lower leg persisting for more than six weeks.

Patients were eligible if they met the following criteria: presence of a chronic ulcer located on the foot, ankle or lower leg; ability to undergo infrared thermographic imaging at baseline; availability of baseline inflammatory markers, including C-reactive protein (CRP) and differential blood count for calculation of the neutrophil-to-lymphocyte ratio (NLR); and availability of 12-week follow-up data or documentation of earlier amputation.

Exclusion criteria comprised acute necrotizing soft-tissue infection, systemic infection requiring immediate operative intervention that precluded thermographic assessment, previous major amputation on the index limb within the preceding three months and missing or unreadable thermographic images.

### 2.3. Thermographic Imaging Protocol

Infrared imaging was performed according to a standardized protocol previously used in our chronic-wound thermography work, with minor adaptations for index development [22]. All measurements were obtained using the same device (FLIR One Pro; Teledyne FLIR LLC, Wilsonville, OR, USA). Emissivity was set at 0.98 for human skin. All measurements were performed by a single trained investigator using a standardized acquisition protocol at baseline, prior to the availability of outcome data. At the time of thermographic assessment, follow-up outcomes and subsequent clinical course were unknown to the investigator, minimizing the risk of outcome-related measurement bias. Thermal images were analyzed immediately at the bedside, and values were recorded prospectively in the study database. Images were not archived for secondary blinded reanalysis, as the protocol was designed for real-time clinical acquisition.

Examinations were carried out in a thermally stable environment with a room temperature of 22 ± 1 °C and a relative humidity of approximately 50%. No directed airflow was allowed towards the limb during imaging. Before acquisition, patients were asked to rest and acclimatize for 10 min to minimize artifacts related to recent changes in limb position or temperature. For each ulcer, the dressing was gently removed, and the wound was left uncovered for 60–90 s to avoid transient temperature artifacts. The thermal camera was positioned at a fixed distance of 30 cm from the wound surface, perpendicular to the ulcer plane.

Two regions of interest (ROIs) were then manually defined on the thermal image: the wound bed (T_bed) and a periwound zone extending 1–2 cm from the wound margin (T_peri). To ensure consistency, the coldest identifiable point within the wound bed was chosen to represent wound-bed temperature, whereas the warmest point within the periwound region—defined as macroscopically intact skin located approximately 1–2 cm beyond the wound edge—was used to represent periwound temperature. From these ROIs, the following variables were derived: mean wound-bed temperature (T_bed, °C), mean periwound temperature (T_peri, °C), and the wound–periwound temperature gradient (ΔT = T_bed − T_peri), representing the local perfusion- and inflammation-related thermal gradient. The pragmatic ROI definition was chosen to reflect the clinically most compromised wound area and the most inflamed periwound zone. This approach prioritizes bedside feasibility, but may introduce operator dependency, which is addressed in the limitations.

Only baseline thermography (day 0) was used for index construction, as early thermal patterns had shown the highest discriminatory potential in prior pilot work [22]. Venous blood samples were collected at baseline as part of routine clinical assessment. From these, the neutrophil-to-lymphocyte ratio (NLR) was calculated using the differential white blood cell count, and C-reactive protein (CRP) concentration was measured.

Clinical follow-up data included the occurrence of major or minor amputation of the index limb, wound healing status at 12 weeks, and length of hospital stay (LOS). Prolonged LOS was defined a priori as a hospitalization longer than seven days, corresponding to the cohort median.

### 2.4. Construction of the Thermal–Inflammatory Index (TI)

To integrate local thermographic data with systemic inflammatory status, we developed a continuous composite index termed the Thermal–Inflammatory Index (TI). The index was constructed from three components: wound-bed temperature (T_bed), neutrophil-to-lymphocyte ratio (NLR) and C-reactive protein (CRP). Each component was standardized using a z-score transformation, and TI was defined as follows:

*TI* = −*z(T_bed)* + *z(NLR)* + *z(CRP)*

where *z(X)* denotes standardization:

$$zX\;=\;\frac{X\;-\;mean(X)}{SD(X)}$$

The sign for T_bed was inverted so that lower wound-bed temperatures, which are indicative of poorer perfusion and worse prognosis, contributed to higher TI values. In contrast, higher NLR and higher CRP, reflecting greater systemic inflammatory activation, were expected to increase TI. In this way, the index was designed to capture the dual physiology of chronic ulcers by combining signals of local perfusion failure and systemic inflammation.

Equal weighting after standardization was deliberately chosen to preserve transparency and clinical interpretability, avoiding regression-derived coefficients that could overfit a modest sample. The formulation reflects a biologically grounded integration of perfusion failure and systemic inflammatory activation rather than a purely data-driven optimization strategy.

### 2.5. TI Risk Groups and Simplified TI Score

For clinical applicability, two derived categorizations were created:

#### 2.5.1. TI Tertiles (Low/Mid/High)

Automatically generated from the distribution of TI in the cohort.

- Low TI: TI ≤ −1.16 (*n* = 27);
- Intermediate TI: –1.16 < TI ≤ 0.93 (*n* = 27);
- High TI: TI > 0.93 (*n* = 28).

#### 2.5.2. TI Score (0–3)

Binary thresholds were applied to the components:

- T_bed low: T_bed < 30.0 °C → 1 point
- NLR high: NLR > 5 → 1 point
- CRP high: CRP > 75 mg/L → 1 point

TI score = 0–3

The binary thresholds were selected as clinically meaningful round values and aligned with ranges commonly reported in the literature, rather than being fully optimized on this dataset. To reduce the risk of overfitting, cut-offs were not tuned to maximize discrimination in the present cohort. Sensitivity analyses using alternative thresholds (T_bed 29–31 °C; NLR 4–6; CRP 50–100 mg/L) were performed to assess the robustness of the TI score.

### 2.6. Outcomes

The primary endpoint of this methodological study was a composite adverse outcome, defined as either major or minor amputation of the index limb or failure to achieve complete wound healing within 12 weeks. Complete wound healing was defined as 100% epithelialization without drainage and without the need for dressing for at least two consecutive weeks. Secondary endpoint included prolonged length of hospital stay (LOS > 7 days).

In additional analyses, we examined the diagnostic performance of TI, T_bed, NLR and CRP using receiver operating characteristic (ROC) curves and area under the curve (AUC) estimates. We also explored risk stratification based on TI tertiles and TI score categories by comparing event rates for the primary and secondary endpoints across these groups.

### 2.7. Statistical Analysis

All statistical analyses were performed using R software, version 4.3.1 (R Foundation for Statistical Computing, Vienna, Austria). The final cohort of 82 patients yielded more than 50 composite events, allowing stable estimation of prespecified multivariable logistic regression models with a limited number of covariates. Continuous variables are presented as medians with interquartile ranges (IQRs) and were compared between groups using the Mann–Whitney U test or Kruskal–Wallis test, as appropriate. Categorical variables are expressed as counts and percentages and were compared using the χ^2^ test or Fisher’s exact test.

The discriminative ability of the Thermal–Inflammatory Index (TI) and its individual components (wound-bed temperature, neutrophil-to-lymphocyte ratio, C-reactive protein) for all study endpoints was assessed using receiver operating characteristic (ROC) analysis, by estimating the area under the curve (AUC) and corresponding ninety-five percent confidence intervals. AUCs and confidence intervals were calculated using the non-parametric DeLong method. Optimal cut-off values for TI and the TI score were identified using the Youden index, and the corresponding sensitivity, specificity, positive predictive value, negative predictive value and likelihood ratios were reported. Differences between AUCs were evaluated using DeLong’s test. Internal validation was performed using bootstrap resampling (5000 iterations). Bootstrap-based 95% confidence intervals were calculated for AUC estimates. For multivariable models, optimism was estimated across bootstrap samples and subtracted from the apparent AUC to obtain optimism-corrected discrimination.

To explore associations between TI and continuous clinical variables, Spearman rank correlation coefficients with ninety-five percent confidence intervals were calculated for TI, its components and selected measures of disease burden, including length of hospital stay and inflammatory markers. Correlation strength was interpreted as weak (absolute ρ < 0.30), moderate (0.30–0.49), or strong (≥0.50).

Logistic regression models were used to evaluate TI as a predictor of the composite adverse outcome and prolonged hospitalization (>7 days) in univariable and multivariable settings. Multivariable models were adjusted for age and high-risk ulcer etiology (diabetic, ischemic, or mixed). Given the exploratory nature of index development, no formal correction for multiple comparisons was applied. A two-sided *p*-value below 0.05 was considered statistically significant.

### 2.8. Ethical Approval

The protocol followed the Declaration of Helsinki and received approval from the Institutional Bioethics Committee of the Jan Kochanowski University Medical College (approval no. 1/2024). All participants provided written informed consent.

## 3. Results

### 3.1. Study Population and Distribution of the Thermal–Inflammatory Index

Eighty-two patients with chronic lower-limb ulcers were included in the analysis. The median age was 72 years (interquartile range 58.3–80.0), and just over half of the cohort were men (57.3%). Diabetes mellitus was present in 58.5% of patients, obesity in 40.2%, and high-risk ulcer etiologies in 45.2%. Venous ulcers were the most common (33 patients, 40.2%), followed by diabetic ulcers (20 patients, 24.4%), ischemic ulcers (9 patients, 11.0%), mixed-etiology ulcers (8 patients, 9.8%), traumatic ulcers (7 patients, 8.5%), and pressure ulcers (5 patients, 6.1%). Empirical systemic antibiotic therapy at admission was initiated in 65.9% of patients.

Baseline infrared thermography showed a median wound-bed temperature of 30.0 °C (interquartile range 28.0–31.6) and a median wound–periwound temperature gradient of −2.15 °C (interquartile range −4.20 to −1.43). Median neutrophil-to-lymphocyte ratio at admission was 5.0 (interquartile range 3.0–8.4), and median C-reactive protein concentration was 75.1 mg/L (interquartile range 26.4–155.8 mg/L). Median length of hospital stay was 7 days (interquartile range 5–13).

By 12 weeks of follow-up, 35.4% of patients achieved complete wound healing, whereas 31.7% required major or minor amputation of the index limb. Overall, 64.6% met the definition of composite adverse outcome, and 48.8% experienced prolonged hospitalization lasting more than 7 days.

The continuous Thermal–Inflammatory Index had a median of −0.29 (interquartile range −1.55 to 1.67), with values ranging from −3.45 to 5.50 and a standard deviation of approximately 2.0. The simplified TI score, constructed as the sum of three binary components (cold wound bed, high neutrophil-to-lymphocyte ratio and high C-reactive protein), ranged from 0 to 3 points and showed an even distribution, with approximately one quarter of patients in each category (24 with a score of 0 points, 14 with 1 point, 23 with 2 points and 21 with 3 points).

### 3.2. Baseline Characteristics and Thermographic Patterns Across TI Tertiles

Patients were stratified into three risk groups according to TI tertiles: low (TI ≤ −1.16), intermediate (TI between −1.16 and 0.93) and high (TI > 0.93). Baseline characteristics according to TI tertiles are summarized in Table 1. Age, sex distribution, prevalence of diabetes and obesity did not differ significantly across TI categories. In contrast, high TI values clustered in patients with more severe etiologies: high-risk ulcers (diabetic, ischemic, or mixed) were present in about one quarter of patients in the low TI group, but in more than half of those in the intermediate and high TI groups, with a statistically significant trend across categories.

Infrared thermography parameters showed a clear, stepwise deterioration with increasing TI. Median wound-bed temperature fell from 32.0 °C in the low TI group to 29.1 °C in the intermediate group and 28.8 °C in the high TI group (*p* < 0.001). In parallel, the wound–periwound gradient became more negative, moving from approximately −1.5 °C in the low TI group to −3.5 °C and −4.1 °C in the intermediate and high TI groups, respectively (*p* < 0.001). These findings indicate progressively colder wound beds relative to the surrounding skin with rising TI values.

Systemic inflammatory markers rose in the same direction. Median neutrophil-to-lymphocyte ratio increased from approximately 2.9 in the low TI group to 4.8 in the intermediate group and 9.7 in the high group (*p* < 0.001). Median C-reactive protein increased from 24.9 mg/L to 61.1 mg/L and 203.9 mg/L across the three strata (*p* < 0.001). Thus, higher TI values captured the convergence of a colder wound bed, greater temperature gradient and more intense systemic inflammatory response.

Indicators of treatment intensity and disease burden also escalated with TI. Empirical systemic antibiotic therapy was used in about one quarter of patients in the low TI tertile, in more than three quarters of those in the intermediate tertile and in almost all patients in the high tertile (*p* < 0.001). Median length of hospital stays increased from 5 days in the low TI group to 8 days in the intermediate group and 13.5 days in the high TI group (*p* < 0.001). The frequencies of amputation, failure to heal within 12 weeks, composite adverse outcome, and prolonged hospitalization all increased sharply across TI categories.

### 3.3. Clinical Outcomes Across TI Score Categories

The ordinal TI score reproduced the risk gradients observed with the continuous TI. Patients with a TI score of 0 points had the most favorable clinical course, with roughly one third experiencing the composite adverse outcome, and the shortest hospital stays (median 5 days). In contrast, patients with TI scores of 2 and 3 points had markedly higher rates of adverse events. Among those with a score of 2 points, more than four-fifths experienced the composite adverse outcome, and over half required hospitalization longer than 7 days. In the highest TI score category (3 points), more than four-fifths of patients met the composite endpoint, and two-thirds had a prolonged hospital stay, with a median hospitalization extending to 14 days. These patterns, summarized in Table 2, support a clear dose–response relationship between TI score and adverse outcomes, suggesting that even this simplified triage scale retains meaningful prognostic information.

### 3.4. Associations Between TI, Inflammatory Markers, and Length of Stay

Spearman correlation analysis demonstrated that the Thermal–Inflammatory Index was strongly aligned with its systemic inflammatory components and moderately associated with resource use. TI showed a very strong positive correlation with the neutrophil-to-lymphocyte ratio (Spearman’s ρ approximately 0.84, *p* < 0.001) and a strong correlation with C-reactive protein (ρ approximately 0.77, *p* < 0.001). Higher TI values were also associated with longer hospital stay, with a moderate positive correlation between TI and length of hospitalization (ρ approximately 0.44, *p* < 0.001).

When individual variables were considered separately, lower wound-bed temperature correlated with more pronounced inflammatory burden and longer stay. Wound-bed temperature correlated negatively with neutrophil-to-lymphocyte ratio (ρ approximately −0.30, *p* = 0.007) and with white blood cell count (ρ approximately −0.33, *p* = 0.002). Its correlation with C-reactive protein was weaker and did not reach statistical significance. In contrast, the length of hospital stay correlated positively with C-reactive protein (ρ approximately 0.29, *p* = 0.007) and neutrophil-to-lymphocyte ratio (ρ approximately 0.32, *p* = 0.004) and negatively with wound-bed temperature (ρ approximately −0.41, *p* < 0.001). These patterns indicate that colder, more inflamed ulcers are typically associated with longer and more complex hospitalizations.

### 3.5. Discriminative Performance of TI and Individual Components

Receiver operating characteristic analysis showed that TI provided discrimination comparable to the best single markers while integrating both local and systemic information. For the composite adverse outcome, the area under the ROC curve for TI was 0.75 (95% confidence interval approximately 0.61–0.87). This performance was very similar to that of wound-bed temperature analyzed in inverted form (area under the curve 0.77, 95% confidence interval approximately 0.66–0.86) and neutrophil-to-lymphocyte ratio (area under the curve 0.74, 95% confidence interval approximately 0.60–0.86), and clearly higher than that of C-reactive protein alone (area under the curve 0.67, 95% confidence interval approximately 0.53–0.79).

**Figure 1:**
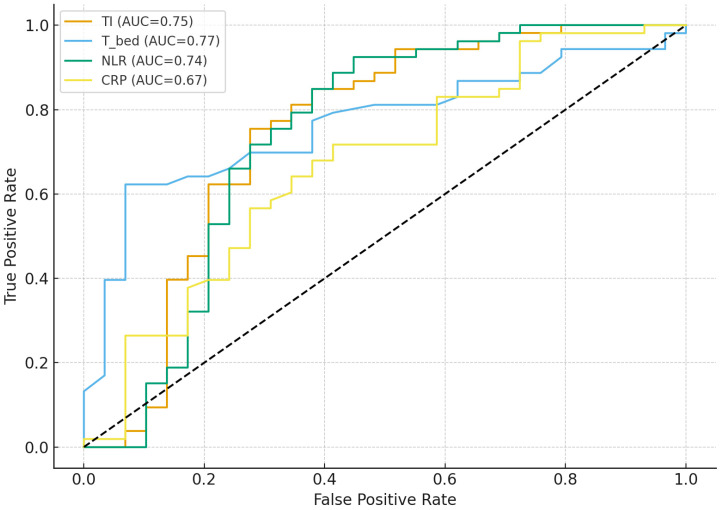
Receiver operating characteristic (ROC) curves for the Thermal–Inflammatory Index (TI), wound-bed temperature (−T_bed), neutrophil-to-lymphocyte ratio (NLR), and C-reactive protein (CRP) in predicting the composite adverse outcome.

For prolonged hospitalization lasting more than 7 days, TI achieved an area under the curve of 0.71 (95% confidence interval approximately 0.59–0.81), again comparable to wound-bed temperature in inverted form (area under the curve 0.73, 95% confidence interval approximately 0.61–0.83) and superior to neutrophil-to-lymphocyte ratio (area under the curve 0.63, 95% confidence interval approximately 0.50–0.75) and C-reactive protein (area under the curve 0.60, 95% confidence interval approximately 0.48–0.72). These results, summarized in Table 3 and illustrated in Figure 1, indicate that TI provides at least the same level of discrimination as the best single marker, while compressing three complementary dimensions—thermal perfusion, neutrophil-predominant inflammation and acute-phase response—into a single continuous variable.

Although detailed reporting of sensitivity, specificity and predictive values for all possible thresholds is beyond the scope of the main text, clinically plausible cut-offs for TI and TI score yielded balanced sensitivity and specificity for identifying patients at high risk of composite adverse outcome and prolonged hospitalization. In the analyses, cut-off values corresponding to the upper TI tertile and TI scores of at least 2 points identified groups in whom adverse outcomes were common, and whose hospital stays were substantially longer.

Bootstrap resampling confirmed the stability of discrimination. For the composite adverse outcome, the bootstrap mean AUC was 0.75 (95% CI 0.62–0.87). For prolonged hospitalization, the bootstrap mean AUC was 0.71 (95% CI 0.59–0.82). In adjusted models (TI + age + high-risk etiology), optimism-corrected AUCs were 0.73 for the composite endpoint and 0.69 for prolonged LOS. Sensitivity analyses using alternative threshold values demonstrated minimal variation in discrimination and event-rate gradients, supporting robustness of the TI score formulation (Table A1).

### 3.6. Multivariable Association of TI with Adverse Outcomes and Length of Stay

To quantify the strength of association between TI and clinically relevant endpoints, logistic regression models were fitted with TI standardized to one standard deviation. In univariable analyses, each one-standard-deviation increase in TI was associated with more than two-fold higher odds of the composite adverse outcome and prolonged hospitalization. Specifically, the odds ratio per one-standard-deviation increase in TI was approximately 2.28 (95% confidence interval 1.29–4.01, *p* = 0.004) for the composite endpoint and 2.20 (95% confidence interval 1.32–3.68, *p* = 0.003) for stays longer than 7 days.

These associations remained robust after adjustment for age and high-risk ulcer etiology. In multivariable models, each one-standard-deviation increase in TI was associated with an adjusted odds ratio of approximately 1.86 (95% confidence interval 1.06–3.25, *p* = 0.029) for the composite adverse outcome and 2.06 (95% confidence interval 1.21–3.50, *p* = 0.008) for prolonged hospitalization. High-risk ulcer etiology independently predicted the composite endpoint with an odds ratio slightly above 3.0 (95% confidence interval just above 1.0–9.4, *p* ≈ 0.044), whereas age showed only a non-significant trend towards higher risk. For prolonged hospitalization, the effect of high-risk etiology was directionally similar but did not reach formal statistical significance. Full numerical details are provided in Table 4.

Together, these findings indicate that TI is not merely correlated with adverse outcomes at the descriptive level but remains independently associated with the composite adverse outcome and prolonged hospitalization, even after accounting for age and high-risk ulcer types. Patients in the highest TI tertile and those with TI scores of at least 2 points form a distinct subgroup characterized by a cold, inflamed ulcer phenotype, intensive antibiotic use, prolonged hospitalization and lack of healing within 12 weeks.

### 3.7. Risk Stratification According to the Thermal–Inflammatory Index

**Figure 2:**
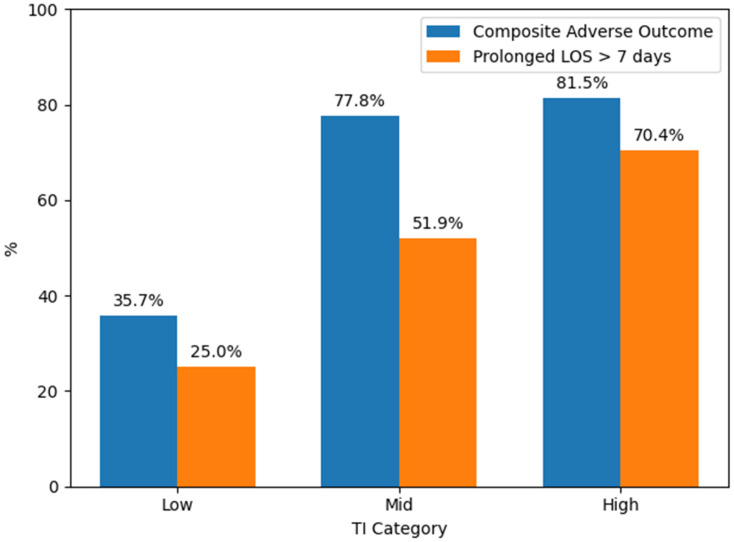
Risk stratification of adverse outcomes according to the Thermal–Inflammatory Index (TI). Bars show the proportions of patients with the composite adverse outcome and prolonged hospital stay (>7 days) across TI tertiles (low, intermediate, high).

Risk stratification according to TI tertiles showed a clear separation of clinical trajectories (Figure 2). Patients in the lowest TI tertile had the most favorable course and the shortest hospital stays, with a median of 5 days. In contrast, patients in the intermediate and high TI tertiles demonstrated progressively worse outcomes, reflecting the accumulation of local thermal impairment and systemic inflammation captured by the index.

In the intermediate TI group, more than three-quarters of patients met the composite adverse outcome. In the highest TI tertile, adverse outcomes were even more concentrated: more than four-fifths failed to heal within twelve weeks or underwent amputation. Length of hospitalization also rose steadily across TI strata, reaching a median of more than 13 days in the highest TI tertile.

These gradients illustrate that TI provides a biologically consistent and clinically actionable stratification of risk, linking the cold–inflamed wound phenotype to markedly prolonged hospitalization and a high likelihood of an adverse clinical trajectory.

### 3.8. Distribution of TI Across Outcome Categories

**Figure 3:**
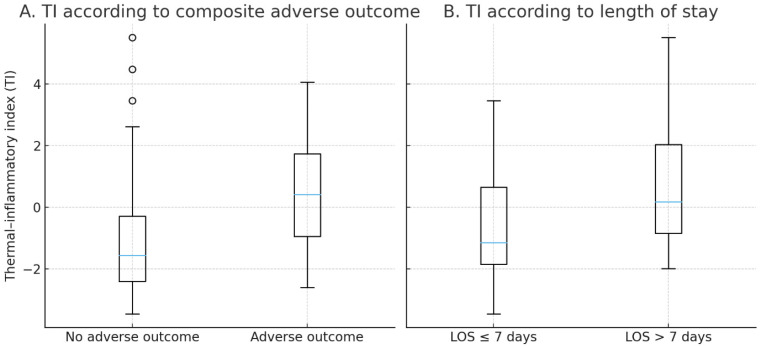
Distribution of the Thermal–Inflammatory Index (TI) across outcome categories. (**A**) TI values in patients with and without composite adverse outcome. (**B**) TI values in patients with short (≤7 days) and prolonged (>7 days) hospital stay.

The distribution of TI values across clinical outcomes is shown in Figure 3. Patients who eventually healed within twelve weeks had distinctly lower TI values, typically falling into the lower and mid ranges of the index. In contrast, TI values in patients who failed to heal or underwent amputation were substantially higher and showed tight clustering in the upper range of the scale.

A similar pattern was seen for hospital stay. Patients with short hospitalizations not exceeding seven days generally had lower TI values, whereas TI values in those requiring longer inpatient care were shifted decisively upward. This separation reinforces the broader observation that TI not only captures baseline severity but also reflects systemic strain and resource use.

Taken together, Figure 2 and Figure 3 show that both the continuous TI and the simplified TI score delineate a reproducible gradient of risk across all major clinical endpoints of interest, including healing failure and prolonged hospitalization.

## 4. Discussion

In this study, we introduce and evaluate the Thermal–Inflammatory Index, a composite biomarker integrating wound-bed temperature with systemic inflammatory markers in patients with chronic lower-limb ulcers of mixed etiology. The index demonstrated moderate discriminative performance, with area-under-the-curve values in the low-to-mid 0.70 s across adverse outcomes, and consistently outperformed CRP while matching or exceeding the prognostic value of NLR. The simplified TI score, consisting of three binary components, produced clear stepwise gradients of risk for failure to heal and prolonged hospitalization. These findings suggest that integrating thermal and inflammatory information into a single construct may provide additional clinical value compared with either domain alone.

TI should be interpreted as a marker of overall disease severity rather than a predictor of a single outcome. Higher TI values reflect the coexistence of impaired local perfusion (lower wound-bed temperature) and systemic inflammatory activation, both of which are biologically linked to tissue hypoxia, increased susceptibility to infection, and delayed wound repair. In this context, TI captures a high-risk physiological profile associated with complicated clinical trajectories and increased healthcare burden.

From a practical standpoint, TI may assist in the early identification of patients who require intensified monitoring and timely escalation of care. Individuals with higher TI scores (≥2) may benefit from prompt reassessment of perfusion status, optimization of wound management, and closer follow-up. Conversely, patients with low TI scores may be suitable for standard outpatient pathways. By facilitating earlier recognition of unfavorable physiological patterns, TI may support more individualized and proactive management strategies.

The rationale for this integration lies in the shared pathophysiology of diverse chronic lower-limb ulcers. Whether primarily diabetic, ischemic, venous, or mixed, many ulcers are shaped by impaired tissue perfusion and chronic inflammatory activation. Microvascular dysfunction limits oxygen delivery and compromises the reparative capacity of fibroblasts, keratinocytes and endothelial cells, while persistent inflammation drives proteolytic activity, oxidative stress and impaired matrix remodeling [1,2,3,4]. Thermographic studies consistently demonstrate that superficial temperature reflects cutaneous blood flow and metabolic activity, with chronically “cold” wound beds and negative wound-to-skin temperature gradients correlating with delayed healing and tissue compromise [16,17,19]. Cooling of the wound surface may reflect poor microcirculatory recruitment or downstream metabolic quiescence, providing a physiologically meaningful signal across ulcer types.

Systemic inflammatory markers contribute complementary information. Elevated NLR reflects a neutrophil-dominant, lymphopenic immune profile associated with impaired healing and an increased likelihood of adverse outcomes in high-risk wound populations, most extensively documented in diabetic and ischemic cohorts but mechanistically relevant beyond these groups [8,9]. CRP correlates with infection severity, poor outcomes after lower-limb interventions and overall inflammatory burden, and experimental evidence indicates that monomeric CRP may directly promote endothelial activation and vascular injury under conditions typical of chronic limb disease [10,11,23]. Although most granular datasets focus on diabetic foot and peripheral arterial disease, the inflammatory pathways they describe—endothelial stress, neutrophil activation, monocyte recruitment—apply broadly across lower-limb ulcer phenotypes.

Our findings align with this mechanistic framework. Patients with higher TI values exhibited lower wound-bed temperatures together with elevated NLR and CRP, a constellation that plausibly reflects combined microcirculatory failure and systemic inflammatory stress. The strong gradients of adverse outcomes observed across TI score categories reinforce the concept that the interplay between local perfusion deficits and systemic inflammation, rather than either mechanism alone, drives risk in chronic ulcers of varied etiology.

Placed in context, the Thermal–Inflammatory Index retains the strengths of established systemic biomarkers while adding information that has previously been difficult to capture outside advanced imaging laboratories. NLR frequently yields AUC values around 0.70 in studies predicting non-healing and amputation in diabetic and ischemic ulcers, supporting its status as a robust, inexpensive inflammatory marker [8,9]. CRP independently correlates with infection severity, vascular instability, and failure of revascularization in infected or ischemic limbs [10,11,13]. Meanwhile, thermography has been explored primarily as a diagnostic or monitoring tool, rather than a prognostic one, and prior reviews highlight the absence of validated thermal indices suitable for risk stratification [17,18,20]. Compared with established wound classification systems (e.g., WIfI, SINBAD), which primarily encode anatomical severity, depth, or ischemia/infection grading, TI focuses on two physiological dimensions—perfusion-related thermal patterns and systemic inflammatory activation. TI is, therefore, complementary and may add a rapid, bedside-accessible layer of risk stratification rather than replacing existing scores.

The translational appeal of the TI score lies in its operational simplicity. By assigning one point for each abnormal component—cold wound bed, elevated NLR and elevated CRP—the score distils multidimensional information into a three-tiered bedside scale. Thermographic acquisition is increasingly feasible using compact smartphone-based devices, while NLR and CRP come from routine laboratory panels available in virtually all care settings [8,9,10,12,13,16,17,18]. In this cohort, patients with a TI score of 0 had very low adverse event rates, whereas those with scores of 2–3 were at substantially increased risk. Such stratification could support early decisions regarding referral, imaging, consideration of revascularization, intensified infection management, or more frequent follow-up. The lower proportion of “failure to heal within 12 weeks” in the highest TI group likely reflects a competing-risk phenomenon: patients with the most severe physiological profile were more frequently amputated earlier, which clinically supersedes prolonged non-healing.

The index may also hold value for monitoring. Interventions designed to enhance microcirculation—such as tibial cortex transverse transport or revascularization procedures—have been associated with increases in limb temperature and improved healing trajectories in selected studies [24,25]. Although our dataset did not include longitudinal thermal measurements, thermal–inflammatory composite metrics may prove useful for tracking response to treatment across ulcer types, particularly if future work links changes in TI to perfusion parameters or molecular biomarkers.

More granular exudate-based biomarker research provides detailed insights into wound biochemistry but remains technically demanding and unlikely to be adopted widely in routine practice [21]. In contrast, the Thermal–Inflammatory Index represents a pragmatic intermediate approach: biologically informed, multimodal in typical clinical environments without specialized laboratory infrastructure. It thus has the potential to complement rather than replace existing clinical scores, laboratory tests and visual wound assessment.

This study was conducted in a single tertiary center and included a modest number of patients, which may limit generalizability. Although the number of outcome events permitted stable estimation of prespecified models, and internal validation was performed using bootstrap resampling, discrimination estimates may still be optimistic. Independent external validation in multicenter cohorts is therefore required before routine clinical implementation.

The cohort comprised ulcers of mixed etiology, reflecting real-world clinical practice but introducing biological heterogeneity that may influence index stability across more homogeneous subgroups. Performance of TI should therefore be further evaluated in etiologically stratified populations, including venous and non-diabetic arterial ulcers.

Thermographic measurements were performed by a single trained investigator using a standardized acquisition protocol, which ensured technical consistency but limited assessment of reproducibility across operators. Because images were analyzed in real time and not archived for independent reevaluation, formal interobserver variability could not be assessed, and reproducibility under varied acquisition conditions remains to be determined. Only baseline thermal and inflammatory measurements were analyzed, but prior thermographic work suggests that temporal trends in temperature may provide additional prognostic information for predicting deterioration or treatment response [18,19,20,22].

Detailed wound morphology parameters were not systematically recorded in this study. Ulcer duration was explored in preliminary analyses but did not demonstrate a significant association with outcomes in this cohort and was therefore not included in final adjustment models. Variables such as ulcer area or depth may provide additional prognostic context and should be incorporated in future studies.

The TI was intentionally constructed using equal standardized weighting to preserve transparency and bedside interpretability. Alternative weighting strategies, including regression-derived coefficients or machine learning approaches, were not explored and may yield incremental gains in discrimination, although potentially at the expense of simplicity and robustness in modest samples.

Finally, the observational design does not establish causality. TI should therefore be interpreted as a pragmatic risk stratification tool reflecting overall disease severity rather than a deterministic predictor of individual outcomes.

## 5. Conclusions

We propose and evaluate the Thermal–Inflammatory Index (TI), a novel biomarker combining local thermal perfusion signals with the systemic inflammatory load. TI demonstrated strong associations with adverse clinical outcomes and provided meaningful risk stratification superior to its individual components. The simplified TI score further supports clinical usability. These findings highlight TI as a promising, low-cost, scalable tool for the early identification of high-risk chronic wounds.

Future research should focus on multicenter validation, the integration of longitudinal thermal data, and the exploration of TI-guided therapeutic algorithms. If confirmed, TI may become a practical element of routine wound assessment, supporting personalized and timely interventions.

## Figures and Tables

**Table 1:** Baseline characteristics according to Thermal–Inflammatory Index (TI) tertiles.

Variable	Low TI (n = 27)	Intermediate TI (n = 27)	High TI (n = 28)	*p*-Value
Age, years	73.5 (62.5–85.0)	72.0 (54.5–75.5)	73.0 (60.0–82.0)	0.438
Male sex, n (%)	9 (33.3%)	22 (81.5%)	16 (57.1%)	0.002
Diabetes mellitus, n (%)	13 (48.1%)	15 (55.6%)	20 (71.4%)	0.299
Obesity, n (%)	7 (25.9%)	14 (51.9%)	12 (42.9%)	0.213
High-risk etiology ^1^, n (%)	7 (25.9%)	16(59.3%)	14 (50.0%)	0.039
Wound-bed temperature, °C	32.0 (30.6–33.2)	29.1 (27.9–30.2)	28.8 (27.2–29.8)	<0.001
Wound–periwound gradient (dT), °C	−1.5 (−1.7–−1.1)	−3.5 (−4.2–−1.6)	−4.1 (−5.1–−2.7)	<0.001
Neutrophil-to-lymphocyte ratio (NLR)	2.9 (2.0–3.7)	4.8 (4.0–6.0)	9.7 (7.7–20.0)	<0.001
C-reactive protein, mg/L	24.9 (10.7–50.1)	61.1 (28.4–101.3)	203.9 (143.8–350.0)	<0.001
Empirical systemic antibiotics, n (%)	7 (25.9%)	21 (77.8%)	26 (92.9%)	<0.001
Length of stay, days	5.0 (4.0–7.5)	8.0 (5.5–11.5)	13.5 (6.0–16.2)	<0.001
Amputation, n (%)	0 (0.0%)	10 (37.0%)	16 (57.1%)	<0.001
Failure to heal within 12 weeks, n (%)	9 (33.3%)	11 (40.8%)	7 (25.0%)	<0.001
Composite adverse outcome ^2^, n (%)	9 (33.3%)	21 (77.8%)	23 (82.1%)	<0.001
Prolonged hospital stay (>7 days), n (%)	7 (25.9%)	14 (51.9%)	19 (67.9%)	0.007

^1^ High-risk etiology = diabetic, ischemic or mixed ulcers. ^2^ Composite adverse outcome = amputation or no healing ≤ 12 weeks.

**Table 2:** Clinical outcomes and length of hospital stay according to TI score (0–3).

TI Score	n	Composite Adverse Outcome ^1^, n (%)	Prolonged LOS > 7 Days, n (%)	Length of Stay, Days (Median [IQR])
0	25	8 (32.0%)	8 (32.0%)	5.0 (4.0–9.0)
1	14	9 (64.3%)	6 (42.9%)	7.0 (5.0–9.8)
2	22	18 (81.8%)	12 (54.5%)	9.5 (5.2–13.0)
3	21	18 (85.7%)	14 (66.7%)	14.0 (7.0–17.0)

^1^ Composite adverse outcome = amputation or no healing ≤ 12 weeks.

**Table 3:** Discriminative performance (AUC) of the Thermal–Inflammatory Index (TI) and individual components for predicting composite adverse outcome and prolonged length of stay.

Outcome	Predictor	AUC	95% CI
Composite adverse outcome ^1^	TI	0.75	0.61–0.87
	T_bed ^2^	0.77	0.66–0.87
	NLR	0.74	0.60–0.87
	CRP	0.67	0.54–0.79
Prolonged LOS > 7 days	TI	0.71	0.59–0.82
	T_bed ^2^	0.73	0.61–0.84
	NLR	0.63	0.50–0.75
	CRP	0.60	0.47–0.72

^1^ Composite adverse outcome = amputation or no healing ≤ 12 weeks. ^2^ For ROC analysis, wound-bed temperature was inverted (−T_bed) so that higher values consistently correspond to higher risk.

**Table 4:** Association of the Thermal–Inflammatory Index (TI) with the composite adverse outcome and prolonged length of stay: univariable and multivariable logistic regression models.

Outcome	Model	Predictor	OR	95% CI	*p*-Value
Composite adverse outcome ^1^	Univariable	TI (per 1 SD)	2.28	1.29–4.01	0.004
Prolonged LOS > 7 days	Univariable	TI (per 1 SD)	2.20	1.32–3.68	0.003
Composite adverse outcome ^1^	Multivariable ^2^	TI (per 1 SD)	1.86	1.06–3.25	0.029
		Age (per year)	1.03	0.99–1.06	0.096
		High-risk etiology ^3^	3.11	1.03–9.35	0.044
Prolonged LOS > 7 days	Multivariable ^2^	TI (per 1 SD)	2.06	1.21–3.50	0.008
		Age (per year)	1.00	0.97–1.03	0.911
		High-risk etiology ^3^	2.09	0.79–5.56	0.139

^1^ Composite adverse outcome = amputation or no healing ≤ 12 weeks. ^2^ Multivariable models adjusted for age and high-risk ulcer etiology. ^3^ High-risk etiology = diabetic, ischemic, or mixed ulcers (vs. low-risk etiologies).

## Data Availability

The data presented in this study are available on reasonable request from the corresponding author. The data are not publicly available due to privacy and ethical restrictions.

## Abbreviations

The following abbreviations are used in this manuscript:

AUC	Area Under the Curve
CRP	C-Reactive Protein
IQR	Interquartile Range
LOS	Length of Stay
NLR	Neutrophil-to-Lymphocyte Ratio
ROI	Region of Interest
ROC	Receiver Operating Characteristic
SD	Standard Deviation
STROBE	Strengthening the Reporting of Observational Studies in Epidemiology
TI	Thermal–Inflammatory Index

## Appendix A

**Table A1:** Sensitivity analysis of alternative TI score thresholds.

Parameter Modified	Threshold	AUC (Composite)	AUC (LOS > 7 Days)	Composite Event Rate (TI ≥ 2)
T_bed	<29 °C	0.750	0.625	0.833
	<30 °C (primary)	0.760	0.663	0.818
	<31 °C	0.740	0.700	0.787
NLR	>4	0.762	0.653	0.804
	>5 (primary)	0.760	0.663	0.818
	>6	0.752	0.660	0.816
CRP	>50 mg/L	0.771	0.669	0.830
	>75 mg/L (primary)	0.760	0.663	0.818
	>100 mg/L	0.761	0.657	0.833

AUC = area under the receiver operating characteristic curve. LOS = length of hospital stay. A composite adverse outcome is defined as amputation or failure to achieve complete wound healing within 12 weeks. Primary thresholds used in the main analysis are marked as “(primary)”.
